# Sustainable Strategy for Algae Biomass Waste Management via Development of Novel Bio-Based Thermoplastic Polyurethane Elastomers Composites

**DOI:** 10.3390/molecules28010436

**Published:** 2023-01-03

**Authors:** Ewa Głowińska, Olga Gotkiewicz, Paulina Kosmela

**Affiliations:** 1 Department of Polymer Technology, Faculty of Chemistry, Gdansk University of Technology, 11/12 Gabriela Narutowicza Street, 80-233 Gdansk, Poland; 2 Institute of Macromolecular Chemistry CAS, Heyrovského náměstí 2, 16200 Prague, Czech Republic

**Keywords:** algae biomass wastes, bio-based thermoplastic polyurethane elastomers, bio-based composites

## Abstract

This work concerns the waste management method of algae biomass wastes (ABW). For this purpose, we prepared bio-based thermoplastic polyurethane elastomer (bio-TPU) composites. Algae biomass wastes are derived from algal oil extraction of *Chlorella vulgaris* and from biomass of *Enteromorpha* and *Zostera marina*. ABWs were used in the bio-TPUs composites as a filler in the quantity of 1, 5, 10, and 15 wt.%. The bio-based composites were prepared via the in situ method. Polymer matrix was synthesized from a bio-based polyester polyol, diisocyanate mixture (composed of partially bio-based and synthetic diisocyanates), and bio-based 1,3 propanediol. In this study, the chemical structure, morphology, thermal and mechanical properties of prepared composites were investigated. Based on the conducted research, it was determined that the type and the content of algae waste influence the properties of the bio-based polyurethane matrix. In general, the addition of algae biomass wastes led to obtain materials characterized by good mechanical properties and noticeable positive ecological impact by increasing the total amount of green components in prepared bio-TPU-based composites from 68.7% to 73.54%.

## 1. Introduction

These days, plastic is one of the most common materials used in almost every branch of industry, including such important ones as medical, construction, automotive, and packaging. In 2020, 55 million tonnes of plastic were produced in Europe, making the plastics market the seventh-largest in the continent. In 2020, only 10.2 million tonnes of plastic were recycled [1]. Globally, 18% of the plastic produced is recycled, 24% is incinerated, and the remaining 58% is landfilled or enters the environment [2]. Most of the plastics available on the market are produced in the processing of raw materials such as crude oil, coal, or gas [1]. These raw materials used in many industries are not renewable and their resources are decreasing year by year. Due to continued population growth as well as economic development, it is assumed that the world’s reserves of fossil fuels will be depleted in the next 40–50 years [3,4]. Another, already mentioned problem with petroleum-derived plastics is their accumulation in the environment. The problem is not the amount of plastic waste alone, but most of all its degradation products. In recent years, contamination with microplastics has been reported in all types of environments around the world and is considered a potential threat to various ecosystems and habitats as well as to human health [5]. Since a large part of society is becoming more and more aware of the problems related to the production of plastics, the pressure to create solutions to minimize these negative aspects is increasing. One of them is the creation of materials that, due to their composition, will be more environmentally friendly and better biodegradable. Among them, we can distinguish plastics made with the use of algae [6]. Algal biomass is divided into microalgae, macroalgae, and cyanobacteria. Algae make attractive candidates for raw materials for the production of plastics. Their advantages include the ability to grow rapidly in various environments, undemanding and relatively cheap cultivation, high accumulation of lipids and proteins in their biomass, as well as the ability to absorb carbon dioxide and treat wastewater. Along with many advantages of algae, their excess in land and water environment generates serious problems. For instance, *Enteromorpha* and *Zostera marina* occur in the Baltic Sea, and their biomass usually can be found on Baltic beaches. Especially during the summertime, the huge amount of algae is a serious interior problem for local society. Products of degradation of *Enteromorpha* and *Zostera marina* blend generate a strong unpleasant odor. In general, along with the accumulation of algae in the coastal areas, costly procedures have to be applied for their removal [7]. What is more, the uncontrolled growth of algae in natural water environments leads to their accumulation. Composting is a simple method to manage the waste derived from algae biomass, but available technology needs improvements [8]. That is why the development of algae waste management is so important these days. For instance, Li et al. prepared bioadsorbents from algae residues for the removal of heavy metals from industrial wastewater [9] while Wang et al. proposed algae-based bioadsorbents for the removal of cadmium ions [10]. This makes bioplastics based on algae a promising and non-toxic alternative that can reduce the consumption of fossil fuels, improve the quality of plastics and minimize the negative environmental impact caused by the excessive use of petrochemical materials [11].

Study on algae-based plastics can be divided into two groups: the development of composite materials (based on polymers of the petrochemical origin or biopolymers, and algae biomass as a filler), and the production of biopolymers or components for their production within the organism of living algae and their subsequent isolating. Unfortunately, in both cases, the efficiency of bioplastics production, as well as the technologies used for this, still require improvement, so algae-based plastics are currently not produced on an industrial scale [12]. Nevertheless, the primary method of producing algae-based plastics is to mix their biomass with petroleum-based plastics or biopolymers. This process has the potential to extend the use of the algae-based composite, as well as provide it with improved chosen properties, e.g., mechanical properties. Currently, polyethylene and polypropylene are the most frequently used polymers as matrices in such composites [13]. In the literature, there are also examples of composite materials based on algae, alginates, algae-based polyols, and polyurethanes as matrices [14]. Nowadays, polyurethanes are the most promising and varied group of polymers with seven distinct types characterised by different properties, from rigid and elastic foams to thermoplastic elastomers and coatings. Due to the large and diverse selection of substrates used in the synthesis of polyurethanes, these materials have a wide range of applications. It also opens the possibility of producing materials with properties tailored to the needs of the user [15]. For this reason, polyurethanes (PU) are used in many industries such as construction, automotive, medical, textile, electronic, packaging, and many others [16].

Algae-based polyurethane composites might be used in different applications, one of which includes algae-based material for the treatment of boron present in oil-produced water described by Syed et al. [17]. The authors determined that the efficiency of boron removal was in the range of 84 to 85% at pH 7.19, depending on the content of the algae. In another example, *Spirulina platensis*, one of the commonly available algae, was used for the preparation of a semi-interpenetrating polymer network composite based on unsaturated polyester resin and polyurethane. The authors determined that algae can participate in forming chemical bonds with the PU component matrix, and causes an increase in thermal stability of obtained bio-composites [18]. It was mentioned previously that algae also constitutes a source for polymer and polyol preparation [19,20]. Phung Hai et al. synthesized novel polyols based on *Nannochloropsis salina* algae and used them in polyurethane synthesis. The starting compounds for algae-based polyols were separated from biomass omega-3 fatty acids which were converted into azelaic acid and heptanoic acid. Bio-based polyester diols are prepared from azelaic acid and ethylene glycol via esterification. This kind of polyols constituted a soft building block for polyurethane foam preparation [20,21]. Algae-based polyols for polyurethane synthesis might be also produced via solvothermal liquefaction of *Enteromorpha* and *Zostera marina* biomass [22,23].

In this work, we present the strategy for algae biomass waste management via development of novel bio-based thermoplastic polyurethane elastomer composites. To our knowledge, up to this point, there is no information about materials based on bio-based thermoplastic polyurethane elastomers filled with *Chlorella vulgaris, Zostera marina,* and *Enteromorpha* as well as their waste derived from algae biomass. Taking that into account in this work, the evaluation of algae-TPU composites and their chemical structure, morphology, and selected mechanical, thermal, and physicochemical properties are presented. Caring for the environment and bearing in mind the 12 principles of green chemistry, environmentally friendly monomers were chosen for the synthesis of bio-based thermoplastic polyurethane elastomers which lead to the design of novel sustainable composites.

## 2. Results and Discussion

### 2.1. Fourier Transform Infrared Spectroscopy (FT-IR)

**Figure 1:**
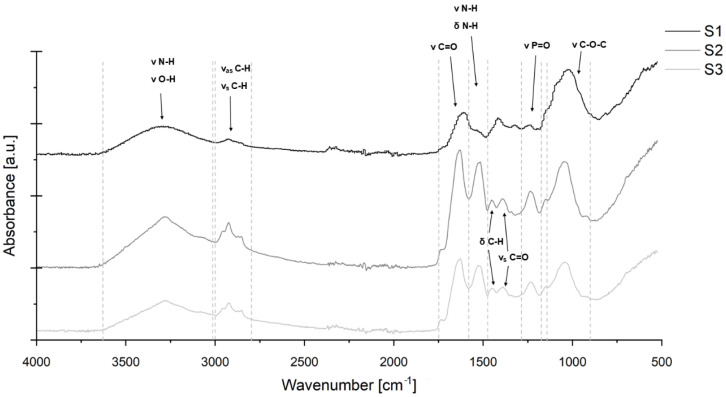
FTIR spectra for algae biomass wastes: S1—*Enteromorpha* and *Zostera marina* blend, S2—*Chlorella vulgaris* after oil extraction, and S3—*Chlorella vulgaris* before oil extraction. In the spectra of algae-based fillers, the specific groups for polysaccharides, lipids, and proteins were recognized in the range of wavenumber 2809–3012 cm^−1^, 1730 cm^−1^, 1425–1477 cm^−1^, 1389 cm^−1^, 980–1100 cm^−1^, and 1191–1356 cm^−1^ [24].

Fourier transform infrared spectroscopy was used for the determination of chemical structure of algae-based fillers, polyurethane matrix, and bio-TPU-ABW composites. In Figure 1, the spectra of *Enteromorpha* and *Zostera marina* blend (S1), Chlorella after oil extraction (S2), and Chlorella before oil extraction (S3) were presented. Prima face, all three spectra are similar to each other, which is the result of the nature of the fillers and the presence of polysaccharides, lipids, and proteins in their structures.

Analyzing FTIR spectra of algae-based fillers (Figure 1)*,* the broad peaks derived from OH and NH groups are revealed in the range of wavenumber from 3029 cm^−1^ to 3639 cm^−1^. This band suggests the occurrence of polysaccharides and proteins in the chemical composition of algae. NH bond also revealed at 1530 cm^−1^ corresponds to bending vibration. At the same wavenumber stretching, vibration of NH also appears. From 2809 cm^−1^ to 3012 cm^−1^, there is the range in the stretching vibration of CH_3_, CH_2_ (symmetric and asymmetric) and CH groups, while bending vibration was assigned in the range of 1425–1477 cm^−1^. Well visible peak at 1730 cm^−1^ attributes stretching vibration of carbonyl bond derived from lipids and polysaccharides. Symmetric stretching vibration of C=O appeared at 1389 cm^−1^. At the range of 980–1100 cm^−1^, a peak of absorption of ether group, C–O–C, was revealed, while at the range of 1191–1356 cm^−1^ stretching vibration for phosphodiester occurred. This discovery also confirms the presence of polysaccharides, lipids, and proteins in the structure of algae [24].

**Figure 2:**
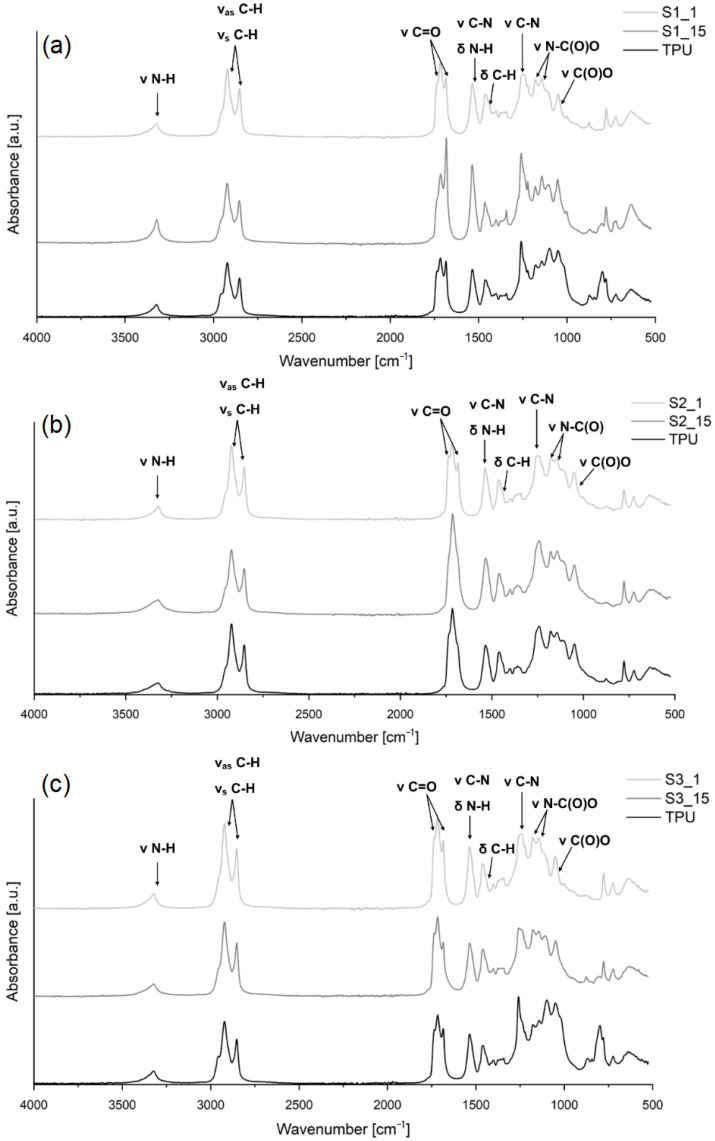
FTIR spectra for TPU composites of each series: (**a**) S1 (bio-TPU based composites containing blend of seagrass *Zostera marina* and *Enteromorpha*); (**b**) S2 (bio-TPU based composites containing *Chlorella vulgaris* after algal oil extraction) and (**c**) S3 (bio-TPU based composites containing neat *Chlorella vulgaris*, before algal oil extraction), which confirmed the presence of urethane groups in the structure of matrix (in all series) by peaks occurring at 3320 cm^−1^, 1730–1685 cm^−1^, 1535 cm^−1^, and 1172–1050 cm^−1^ characteristic for NH, C=O, CN, and N-C(O)O bonds and the absence of stretching vibration of NCO at 2250 to 2270 cm^−1^ [27,28].

The analysis of FTIR was also conducted in order to confirm the chemical structure of bio-based polyurethane matrix and algae-based composites. In Figure 2a–c, registered FTIR spectra are presented. As a matter of priority, the creation of urethane groups in the structure of the matrix (in all series) was confirmed by peaks occurring at 3320 cm^−1^, 1730–1685 cm^−1^, 1535 cm^−1^, and 1172–1050 cm^−1^ characteristic for NH, C=O, CN, NH, and N-C(O)O bonds. Further confirmation of the presence of the urethane group is the absence of the NCO group at wavenumber ranging from 2250 to 2270 cm^−1^ [25,26].

If we look closely at particular absorptions, we can notice that the stretching vibration of NH occurred at ca. 3320 cm^−1^ and was registered as a single peak, and the wavenumber suggests that most of the NH groups are hydrogen-bonded [27]. At 1529 cm^−1^, two vibrations were assigned related to the part of the urethane group -CN-H: bending vibration for NH and stretching vibration for CN. The peaks at 1150–940 cm^−1^ are associated with symmetric stretching of N–C(O)O and stretching vibration of C–O–C [27]. Analysing the carbonyl group, we consider a multiplate peak occurred ranging from 1685 to 1730 cm^−1^ with the three maxima at 1685 cm^−1^, 1716 cm^−1^ and 1730 cm^−1^. The first maximum corresponds to the carbonyl group in the allophone group derived from the partially bio-based isocyanate, the second and third maxima are related to hydrogen-bonded and free carbonyl groups in urethane, respectively [29].

In the structure of bio-TPUs and their ABW-based composites, methyl group, methylene and methine group are present. In the FTIR spectra, the mentioned groups are revealed as stretching vibration at the range of wavenumber from 2809 cm^−1^ to 3012 cm^−1^ and bending vibration of CH_3_ and symmetric deformation of CH_2_ in the range from 1430 to 1459 cm^−1^ [27,28].

### 2.2. Optical Microscopy

**Figure 3:**
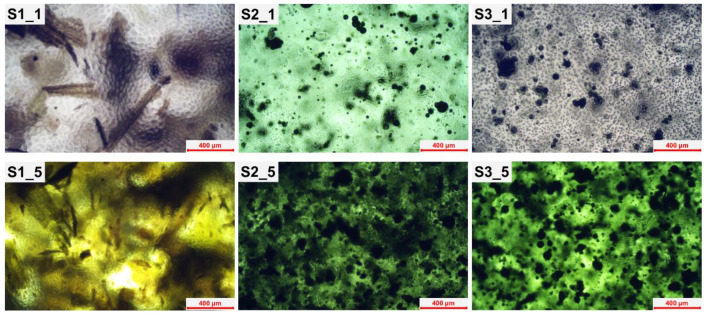
Optical micrograph of algae-TPU-based composites. Series S1 filled with *Enteromorpha*–*Zostera marina* blend, series S2 contained *Chlorella vulgaris* waste—algae after oil extraction and S3 series *Chlorella vulgaris* before oil extraction.

Optical micrographs (Figure 3) show representative examples of all series of obtained bio-TPU-ABW composites, filled with 1 and 5% of algae. Analysing the optical micrographs, it can be seen that the structure of the used algae and their distribution in the bio-TPU matrix differ depending on the used filler. *Chlorella vulgaris*’ particles are fairly regular, spherical in shape, while the *Enteromorpha–Zostera marina* blend filler contains irregular, elongated fragments. The size, chemical composition and morphology of algae-based fillers might be the reason of the distribution of filler in the bio-TPU matrix and their tendency for the formation of particle agglomeration. Considering algae-TPU composites containing *Chlorella vulgaris* (see series S2 and S3), we can observe filler agglomeration which is even more visible in the case of S3 series (*Chlorella vulgaris* before oil extraction). When we take into consideration the *Enteromorpha–Zostera marina* blend, lower tendency of the filler agglomeration can be observed that can be a result of poor interaction between algae particles. Overall, it was determined that the fillers were mostly regularly dispersed in the matrix. Differences between the structure and behaviour of the filler in the polymer matrix can greatly affect the properties of the composites.

### 2.3. Thermogravimetry

**Figure 4:**
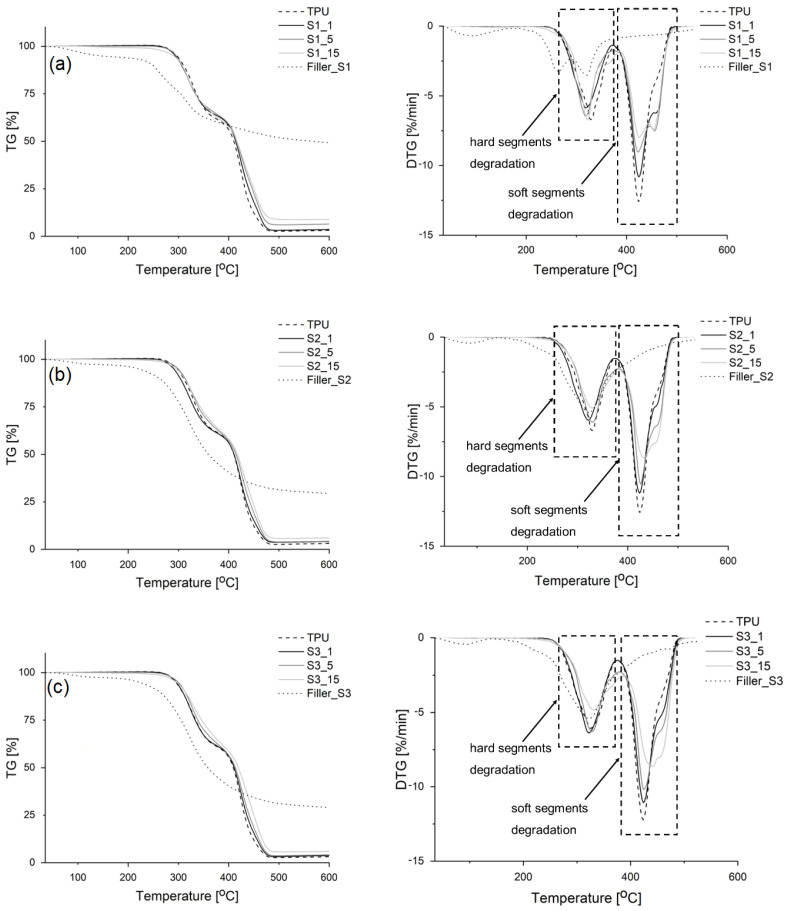
TG and DTG as a function of temperature for bio-TPU-ABW composites: (**a**) S1, (**b**) S2 and (**c**) S3.

Thermogravimetric analysis was carried out for thermoplastic polyurethanes and composites with algae biomass waste with 1, 5 and 15% filler in the bio-TPU-based matrix. TGA analysis was performed in order to investigate thermal stability of prepared materials and the influence of ABW on bio-TPU matrix. The results of the TGA analysis were demonstrated on the TG and DTG curves (Figure 4a–c). The temperatures of 5, 10, and 90% weight loss were determined. An ash residue as a weight % was assigned at a temperature of 600 °C.

Taking into consideration algae based-fillers, their thermal stability depends on the algae source. *Enteromorpha–Zostera marina* blend (filler S1) began to decompose at the lowest temperature of ca. 143.1 °C and its degradation occurred in two stages. High content of residue (49.2% at 600 °C) suggests the high content of polysaccharides and inorganic compounds [30]. Thermal decomposition of *Enteromorpha–Zostera marina* blend occurred at a narrower temperature range compared to *Chlorella vulgaris*, and presumably as a consequence of high content of cellulose [22].

Fillers based on biomass wastes derived from *Chlorella vulgaris* (S2 and S3), according to the literature [31], in N_2_ atmosphere decomposed in one main stage. Temperature of the beginning of degradation (T_5%_) for biomass waste of *Chlorella vulgaris* was noticed at 220.8 °C while for neat *Chlorella vulgaris* it was noticed at 223.5 °C. If we look closer at the DTG curves, a small peak in the range of 50 to 180 °C is visible, which indicates the breakdown of lipids and proteins. Main peak on the DTG curve of the degradation involves thermal decomposition of carbohydrates, protein, and lipids [32].

If we take into consideration the influence of algae biomass waste on thermal stability of bio-TPU-based composites, it is noticeable that in most cases the thermal decomposition process in these materials started at almost the same temperature that in the case of the bio-TPU matrix. In general, it can be stated that the increase in *Chlorella vulgaris* as an ABW filler caused a slight improvement in thermal stability of bio-TPU-based composites. Materials obtained with the use of *Enteromorpha–Zostera marina* blend filler were characterized by a mildly lower thermal stability. Based on the determined T_50%_ parameter, it could be observed that with the increase in the ABW content (all types) in the polymer matrix, the temperature at which the material underwent thermal decomposition increased. It is probably caused by the presence of lipids in the algae biomass. The ash residue increased correspondingly with the increase in algae biomass wastes in the composites.

As mentioned above, bio-TPU- and ABW-based composites degrade in two main steps. If we consider DTG curves presented in Figure 4, it can be seen that each peak occurred as a multiplate. The addition of algae, which degraded at the temperature of about 350 °C [33], also caused the broadening of the first peak present in the DTG curves. Additional shoulder in the DTG diagrams in the temperature range of 442–458 °C corresponds to the decomposition of organic compounds contained in algae, with particular emphasis on fatty acids and bio-based polyols [31,32].

Analysing Table 1, it could be observed that bio-based thermoplastic polyurethane elastomers used as a composites’ matrix are characterized by thermal stability of up to 296.8 °C related to the temperature of a 5% weight loss (T_5%_). After reaching this temperature, the material began to undergo the process of thermal decomposition. For all TPUs, it was observed that their thermal decomposition took place in two stages resulting from segmented structure of bio-TPU, and was revealed in the DTG diagrams as two separated peaks. The first stage of decomposition begins at the temperature of 325–329 °C, which corresponds to the thermal dissociation of hard segments, while the second—at 423–424 °C, which is related to the degradation process of soft segments composed of the polyester polyol chains [34,35]. Increase in algae based-filler in the bio-TPU matrices caused increasing of residue at 600 °C.

**Figure 5:**
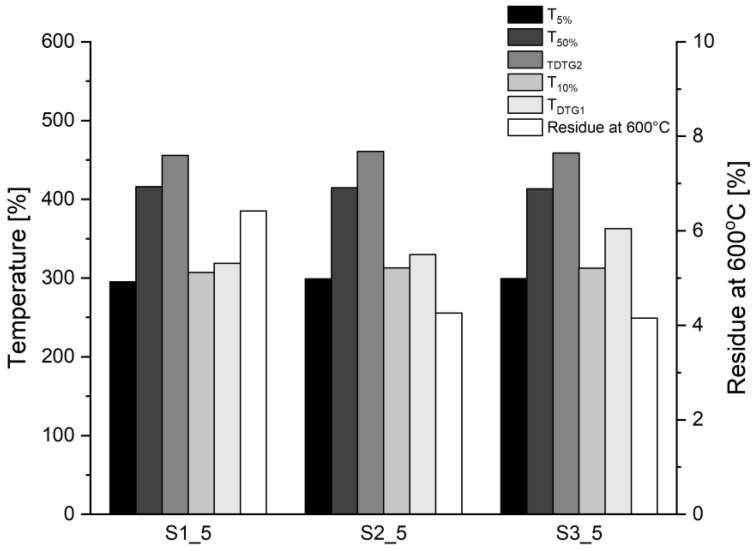
Comparison of T_5%_, T_10%_, T_50%_, T_DTG1_, T_DTG2_, and residue at 600 °C for bio-TPU–ABW composites containing 5 wt.% of the ABW fillers.

To provide a more complete understanding of the type of algae waste biomass influence on the thermal properties of composites, the comparison of selected parameters for bio-TPU-ABW composites containing 5 wt.% of the fillers was performed. In Figure 5, the bar chart with parameters T_5%_, T_10%_, T_50%_, T_DTG1_, T_DTG2_, and residue at 600°C is presented. Analysing Figure 5, it can be seen that the type of ABW filler has a main impact on TDTG1 and residue at 600 °C and might be related with the chemical composition of ABW fillers and content of polysaccharides and inorganic compounds.

### 2.4. Dynamic Mechanical Analysis

DMA analysis was conducted in order to examine the viscoelastic behaviour of obtained bio-TPU–ABW composites at the broad range of temperatures from −100 °C to +100 °C. In general, storage modulus provides the information about solid-like (elastic) behaviour of polymers, including their stiffness. The loss modulus reveals the information about liquid-like behaviour of the material, their viscous response which is associated with the sample’s heat energy dissipation. The complex modulus as ratio of loss and storage modulus is known as a loss factor or damping factor, indicating the material’s resistance to deformation [36,37].

**Figure 6:**
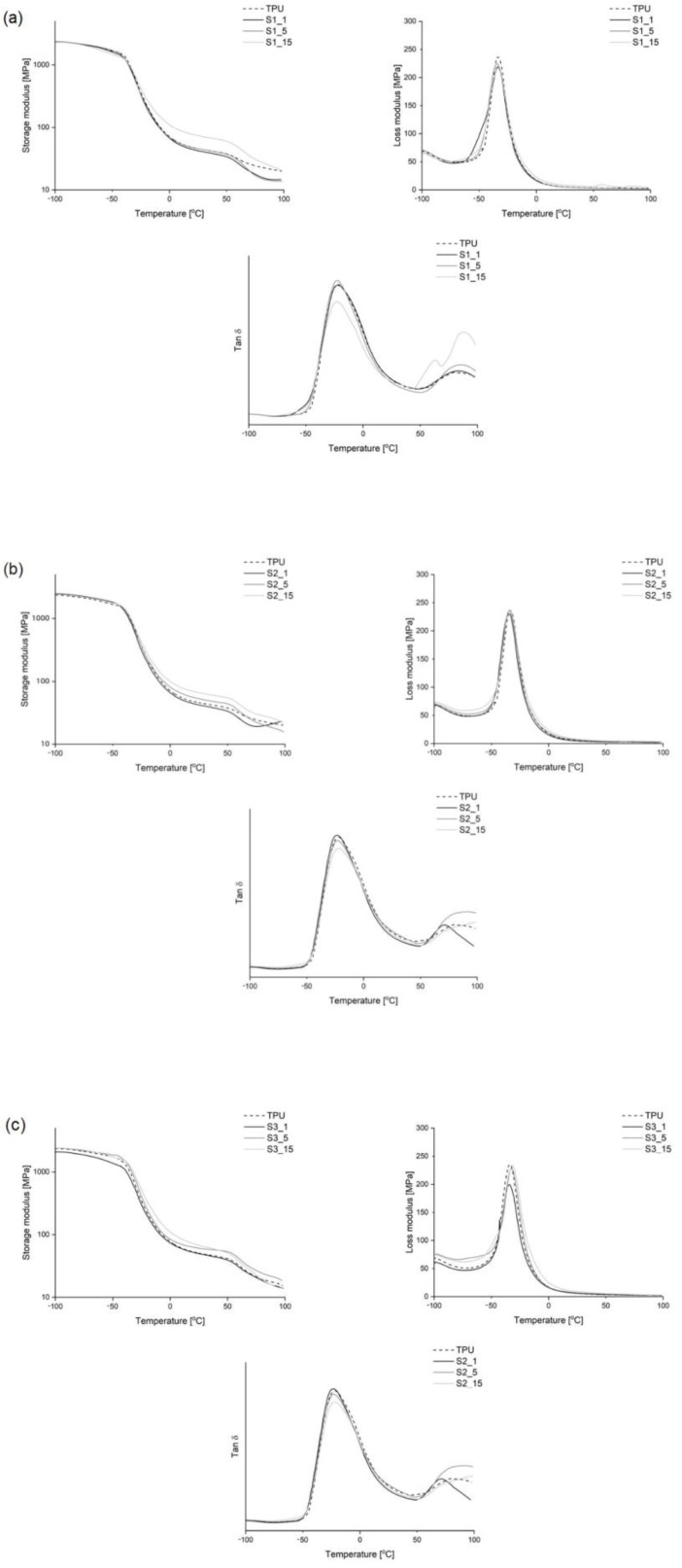
The storage (E’), loss modulus (E’’) and tanδ in the function of temperature for bio-TPU-ABW composites: (**a**) series S1, (**b**) series S2 and (**c**) series S3.

Bio-TPU matrix and composites filled with biomass waste of *Chlorella vulgaris* and *Enteromorpha–Zostera marina* blend (1, 5 and 15% filler in the matrix) were subjected to dynamic mechanical analysis. Based on this, the dependencies of the dynamic modules—storage (E’) and loss (E’’)—on temperature were determined and plotted in Figure 6a–c. Additionally, based on the loss factor (tanδ), the glass transition temperatures (T_g_) of the materials were determined (Table 2).

Analysing the storage modulus of all series of the bio-TPU–ABW composites, the influence of type and amount of algae biomass waste is clearly visible. Higher loading of algae biomass waste in the bio-TPU matrix caused slightly enhanced elastic properties of prepared composites. Taking into account the course of the curve of storage modulus revealed the segmented structure of bio-TPU matrix and their thermoplastic behaviour. The region of glass transition (sharp decrease in E’) of soft segments ranges from ca. −50 °C to 0 °C and in the case of hard segments ca. 50–100 °C. Above this temperature, bio-TPU matrix is in the plastic state and begins to flow. Based on storage modulus curves, the reinforcement effect of bio-TPU matrix by the ABW incorporation was revealed. The storage modulus at room temperature at 25 °C is higher for bio-TPU–ABW composites in comparison to bio-TPU matrix caused by interaction between algae-based filler and bio-TPU matrix, and leads to better stress transfer at the interface of the composites [38,39]. Considering the dependence of the loss modulus on temperature, it should be noted that in the case of S1 and S2 composites it was lower than in that of the bio-TPUs matrix, only the S2 samples obtained higher values. This means that in the case of composites filled with *Chlorella vulgaris* after oil extraction, more energy was lost due to its dissipation [40].

With increase in ABW filler in composites, a decrease in loss factor was observed. This result indicates stress transfer from bio-TPU-based matrix to the ABW-based filler and thereby limits the chain mobility in the matrix, which implies that the friction of the intermolecular chain is reduced [41]. Almost in most cases of bio-TPU-ABW composites, the addition of the ABW filler caused the glass transition temperature of soft segments to shift towards higher values (determined based on loss factor curves). The T_gSS_ difference was insignificant as it was less than 1 °C. On this basis, it can be concluded that the addition of ABW filler had a negligible effect on the glass transition temperature of bio-TPU–ABW composites. Analysing both storage modulus and loss factor curves, the glass transition of hard segments in the range of 50–100 °C was observed.

**Figure 7:**
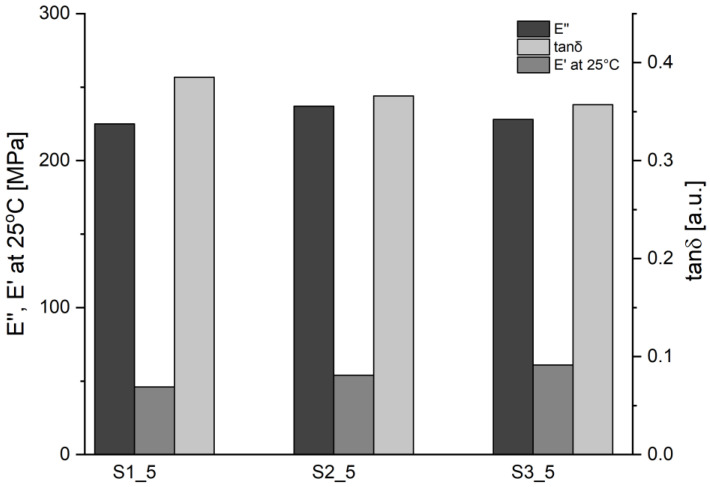
Comparison of such E’’, E’ at 25 °C and tanδ for bio-TPU–ABW composites containing 5 wt.% of the ABW fillers.

Comparison of the dynamic mechanical properties such E’’, E’ at 25 °C and tanδ of bio-TPU–ABW composites with constant amount (5 wt.%) is presented in Figure 7. The influence of type of the ABW fillers is visible in the storage modulus at 25 °C, which indicates the strengthening effect of the matrix by filler addition.

### 2.5. Tensile Properties and Hardness

**Figure 8:**
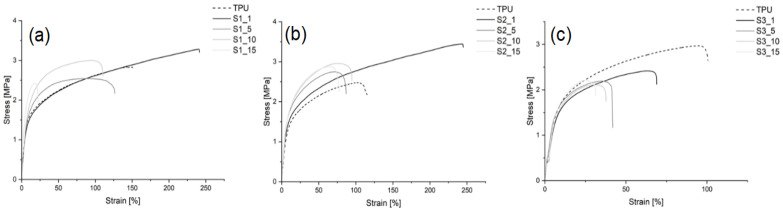
Stress-strain curves for the tested bio-TPUs matrices and bio-TPU–ABW composite series (**a**) series S1, (**b**) series S2 and (**c**) series S3.

Tensile strength (TS_b_) and elongation at break (ε_Break_) were determined for bio-TPU matrix and bio-TPU–ABW composites in order to examine the mechanical properties of obtained materials. The tensile test results are shown in Table 3 and Figure 8.

Based on theory, the reinforcement effect caused by algae biomass waste addition should portray increases in tensile strength of bio-PTU–ABW composites with increase in ABW filler content. Analysing the obtained data (Table 3), it can be seen that tensile properties depend on the type of algae. Bio-TPU–ABW composites containing filler S1 and S2 are characterized by enhanced tensile strength compared to bio-TPU matrix, which was also confirmed by the DMA results discussed earlier. With increase in the S1 and S2 fillers, a slight increase in tensile strength and decrease in elongation at break were observed (excluding composites coded S1_5 and S1_15 which might be caused by a random distribution of the filler in the bio-TPU matrix). Nevertheless, an increase in filler content may have limited the mobility of the bio-TPU matrices that resulted in lower elongation at break for bio-TPU–ABW composites compared to bio-TPUs matrices (see Figure 8).

Considering tensile strength and elongation at break registered for bio-TPU–ABW composites, series S3, it should be noted that for both parameters—tensile strength and elongation at break—the values characterizing the composites were much lower than those corresponding to the bio-TPU matrix. Moreover, the presented data show that the TS_b_ and ε_Break_ decreased with the increase in the neat *Chlorella vulgaris* content (Table 3).

Additionally, the trend observed in S3 could be noticed again, i.e., with an increase in the S3 filler content in the bio-TPU matrix, the maximum elongation at break of the composite decreased, which is a common phenomenon in filled polymers and is caused by a reduction in the elasticity of the rigid interphase between the filler and the matrix, which increases the brittleness of the material [42].

In general, composites produced based on natural fibres often exhibit poorer mechanical properties in terms of tensile strength and elongation at break. This is confirmed by the results obtained for S3, containing neat *Chlorella vulgaris* (before oil extraction). This phenomenon can be explained by the differences in the polarity bio-TPU matrix and the S3 filler, affecting the incompatibility of these two components. The different nature of these ingredients can negatively affect the level of adhesion between the filler and the matrix and also enhancement of the filler agglomeration. It is worth noting that the higher the filler content, the greater the probability of its agglomeration in the matrix what was noticed in the micrographs (Figure 3c). In addition, the higher content of filler fibres in the matrix also increases the sorption properties of the composite, causing an increase in moisture within it. These phenomena negatively affect the tensile strength and elongation at break of composites. Therefore, with an increase in the amount of filler, these properties are weaker, which was confirmed by the conducted studies [43,44].

The results of hardness of bio-TPUs and bio-TPU–ABW composites are reported in Table 3. Prima face, it can be seen that in almost all cases hardness increases with an increase in algae-based fillers. Many different factors influence the hardness (e.g., morphology, chemical interaction or tendency to phase separation) which results from the intermolecular cohesive forces between the polymer chains and/or polymer chains and filler [45].

In the case of prepared bio-TPU–ABW composites, hardness depends mainly on the algae-based filler type, distribution of the filler in the polymer matrix and interaction between filler and matrices. Analysing composite series S1, it can be stated that bio-TPU–ABW composites containing seagrass wastes such as *Enteromorpha–Zostera marina* blend were characterized by higher hardness compared to bio-TPU matrix. However, the dependence between hardness and fillers content has not been observed. It is probably a result of the random distribution of S1 filler in bio-TPU matrix.

For hardness of composite series S2 and S3 where the filler was *Chlorella vulgaris* (wastes and neat), the increase in hardness was visible with the increasing content of algae in the composite. This indicates some strengthening effect of the bio-TPUs matrices, and good adhesion between the matrix and the filler. Bio-TPU–ABW composites containing algae biomass wastes (S2) each show a slightly higher hardness than those filled with *Chlorella vulgaris* before extraction (S3). If we take into account the values of hardness, we can classify the obtained bio-TPU and bio-TPU composites as thermoplastic elastomers [46].

**Figure 9:**
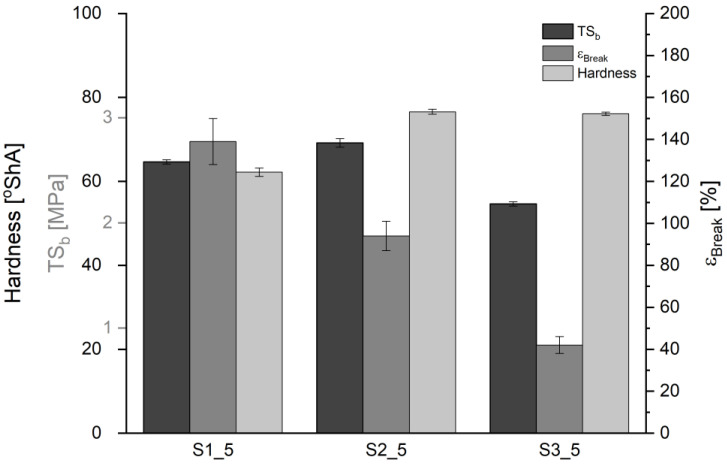
Comparison of T_Sb_, ε_Break_, and hardness for bio-TPU–ABW composites containing 5 wt.% of the ABW fillers.

In Figure 9, the comparison of the TSb, εBreak, and hardness for bio-TPU–ABW composites containing 5 wt.% of the ABW fillers is provided. Analysing mechanical properties, the influence of the type of ABW fillers was revealed, especially in tensile strength and elongation at break. The strengthening effect (the highest of TSb and hardness) of the bio-TPU matrices was noticed in the case of composites containing algae waste—*Chlorella vulgaris* after extraction.

### 2.6. Water Absorption

**Figure 10:**
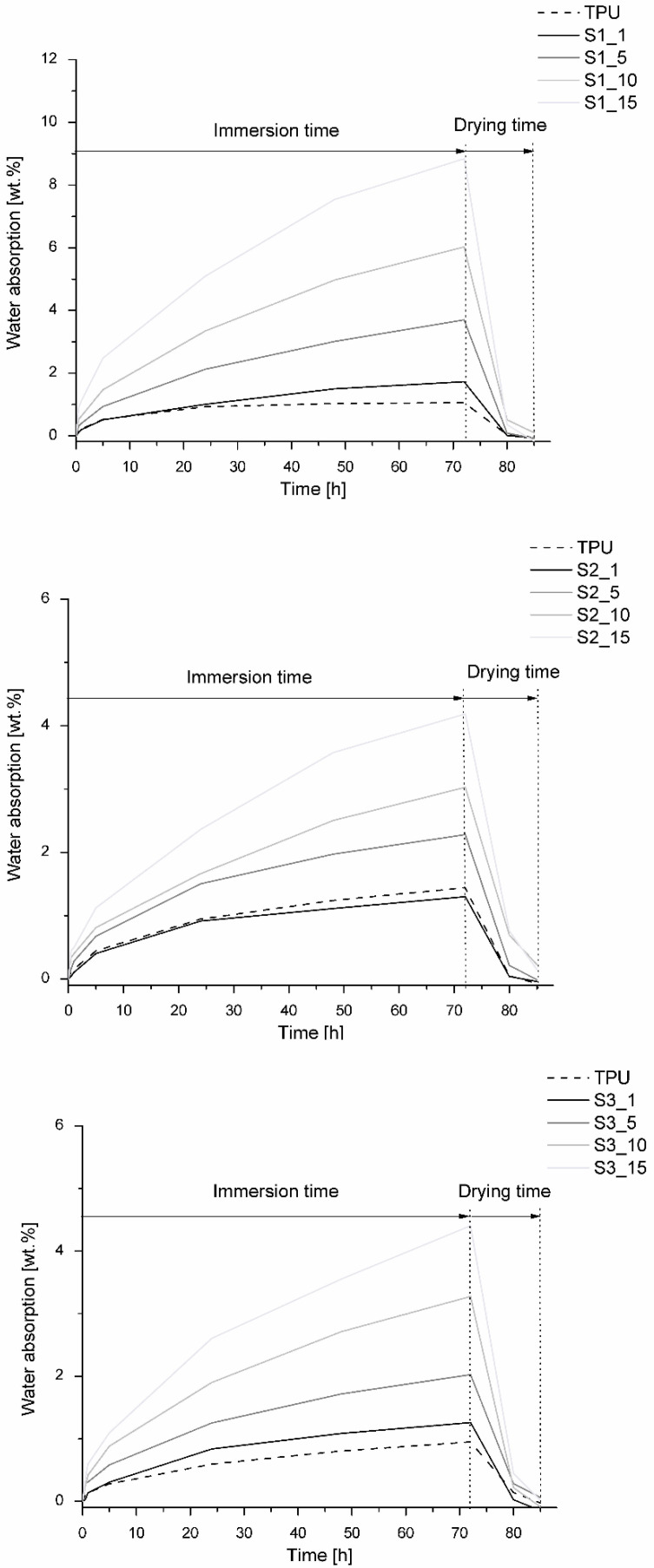
Water absorption graph for bio-TU matrices bio-TPU–ABW composite series S1, S2, and S3 during sample immersion in water and after drying.

Water absorption analysis was carried out for bio-TPU matrices and bio-TPU–ABW composites. Results are presented as water content vs immersion time graph in Figure 10, which shows the percentage of water absorption of bio-TPU–ABW composites. Algae as a filler is highly moisture-sensitive, which can be revealed in properties of algae-based composites. That is why higher water absorption for bio-TPU–ABW composites was suspected. Overall, water absorption of the natural filler may be attributed to the presence of the hydroxyl groups in the cellulosic structures that form a hydrogen bond with the molecule of water [38]. Analysing Figure 10, it is clearly shown that that assumption was confirmed. With an increase in the algae-based filler content in the bio-TPU–ABW composite, the water absorption of the material increases. The bio-TPU–ABW composite series S2 and S3 (filled with waste and neat *Chlorella vulgaris*) were characterized by lower water absorption, while for bio-TPU–ABW composites filled with *Enteromorpha–Zostera marina* it was almost twice as high. It can be a result of high content of polysaccharides in the structure of seagrass, and on the other hand a result of morphology of the filler and obtained composites.

It should be noted that the differences in water absorption between the two series of *Chlorella vulgaris*-based composites were unnoticeable. Regardless of the type of algae-based filler, in all cases of bio-TPU–ABW composites filled with 1% of filler the difference in absorption between composites with different fillers was smaller.

## 3. Materials and Methods

### 3.1. Materals

Bio-based polyester polyol with the trade name Priplast 3238 was obtained from Croda, United Kingdom. Aliphatic hexamethylene diisocyanate (HDI) and partially bio-based aliphatic isocyanate polymer—Tolonate™ X FLO 100 (X FLO) were purchased from Vencorex, France. Bio-based 1,3-propanediol (trade name Susterra^®^) used as chain extender was produced by DuPont Tate & Lyle BioProducts, Loudon, Tennessee, USA. Dibutyltin dilaurate was obtained from Sigma Aldrich (division in Poland). Three types of algae were used as a filler—*Chlorella vulgaris* (Holistic Chlorella, Sweden)—before and after prior extraction of algal oil using hexane, and a blend of *Zostera marina* and *Enteromorpha* (mass ratio 9:1), both collected in the Gulf of Gdansk (Baltic Sea) and subjected to solvothermal liquefaction beforehand. The *Chlorella vulgaris* was in the form of a powder, whereas the second filler came in the form of flakes.

### 3.2. Matrix Preparation

**Scheme 1:**
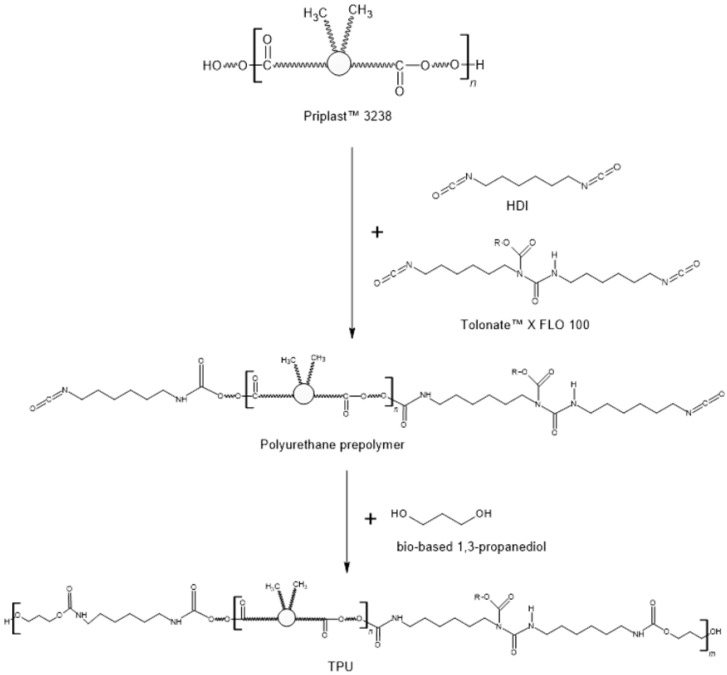
Scheme of the synthesis of bio-based thermoplastic polyurethanes via the prepolymer method. The first stage—synthesis of the prepolymer by the reaction of diisocyanate mixture with polyol, and the second stage—extension of urethane prepolymer chains by using bio-based 1,3-propanediol.

Bio-based thermoplastic polyurethanes elastomers were obtained using the prepolymer method (see Scheme 1). In the first stage, the prepolymer was synthesized using polyester bio-polyol, with an average molecular weight of 2000 g/mol and a mixture of aliphatic diisocyanates composed of 75 wt.% of HDI and 25 wt.% of X FLO. The obtained urethane prepolymer had 6 wt.% of unreacted isocyanate groups (determined by the titration method according to ISO 14896 standard). In the second stage, the prepolymer’s chain extension reaction was carried out using bio-based 1,3-propanediol. Dibutyltin dilaurate was used as a catalyst. The chain extension reactions were performed with the molar ratios of [NCO]/[OH] groups of 0.95 (composite series S3) and 1.0 (composite series S1 and S2). The bio-based TPUs were cured at 100 °C for 24 h in a laboratory oven.

### 3.3. Composites Preparation

Prior to use, all the fillers were dried at 70 °C for 4 h in a laboratory oven. The composites were produced by dispersing the filler in the prepolymer (portions of 40 g) by manual stirring with a rod. Next, bio-based 1,3-propanediol was added and then subjected to a chain extension reaction, degassed, and poured into heated moulds at a temperature of ~65 °C. After the polymer solidified, algae–TPU composites were placed in a laboratory oven at 100 °C for 24 h to cure. Algae–TPU composites with 1, 5, 10, and 15 wt.% of algae were prepared and divided into three series of materials. Composites with seagrass *Zostera marina* and *Enteromorpha* were coded as S1, materials with algae waste based on *Chlorella vulgaris* after algal oil extraction were coded as S2, and composites with *Chlorella vulgaris* before algal oil extraction (reference filler) were coded as S3. Codes of samples are given in Table 4.

### 3.4. Characterization of the Bio-Based Composites

#### 3.4.1. Fourier Transform Infrared Spectroscopy (FT-IR)

Using infrared spectroscopy with Fourier transform, the chemical structure of the fillers, obtained bio-based thermoplastic polyurethanes, as well as algae–TPU composites were investigated. The tests were performed with the Nicolet 8700 apparatus, using the FTIR-ATR technique. The measurement was performed under normal pressure and at room temperature. The apparatus performed 64 scans per sample, its resolution was 4 cm^−1^, and the wavenumber range was 500–4000 cm^−1^.

#### 3.4.2. Optical Microscopy

The morphology of the obtained algae–TPU composites was examined using optical microscopy. For this purpose, the Delta Optical LH100-1 microscope was used. The microscope image was captured with a digital camera connected to the Touptek-MTR3CMOS-20000KPA microscope. Photos of the samples were taken at five times magnification, using the X-ray technique.

#### 3.4.3. Thermogravimetry (TGA)

The thermogravimetric analysis of algae biomass wastes and bio-TPU–ABW composites was performed with the use of the NETZSCH TG 209F3 apparatus. The samples were heated under a nitrogen atmosphere at a rate of 10 °C/min, in the temperature range from 35 to 800 °C. The mass of each sample was about 10 mg.

#### 3.4.4. Dynamic Mechanical Analysis (DMA)

DMA Q800 apparatus Q800 analyzer (TA Instruments) was used for dynamic mechanical analysis. Measurements were taken following ASTM D4065:2012. For measurements, bio-TPU-based matrix and bio-TPU–ABW composites with dimensions of 18 × 10 × 3 mm were used. The samples were subjected to cyclic bending with a frequency of 10 Hz in the temperature range from −100 °C to 100 °C with a temperature change rate of 3 °C/min.

#### 3.4.5. Tensile Properties

The tensile strength measurement under static conditions was carried out with the Zwick/Roell Z020 testing machine. During the test, dumbbell-shaped specimens with a measuring distance of 25 mm were tested at a cross-head speed of 100 mm/min. Measurements were carried out at room temperature according to PN-EN ISO 527-1: 1998. The tensile strength (TS_b_) and elongation at break (ε_Break_) were calculated as the mean of five measurements.

#### 3.4.6. Hardness

The determination of the hardness of bio-based thermoplastic polyurethane elastomers and bio-TPU–ABW composites was performed using a digital Shore A hardness tester by Zwick/Roell according to the PN-EN ISO 868: 2005 standard. For each sample, 6 measurements were taken.

#### 3.4.7. Water Absorption

The water absorption determination was performed by examining the weight change of 10 × 10 mm samples, which were placed in distilled water at room temperature for a specified time. Three samples of bio-TPU and algae-TPU-based composites with 1, 5, 10, and 15% algae content from each series of materials were tested. Before testing, the samples were placed in a laboratory oven at 60 °C for 24 h. Plates were weighed before immersion in water and after 0.5, 1, 5, 24, 48, 72 h. After determining the absorption, the samples were left in the air for three days, after which their weight was measured again. Then, they were placed in an oven at 60 °C for one hour, after which the last mass measurement was made. The water absorption was calculated according to Equation (1). The test was performed in accordance with PN-EN ISO 62: 2008 standard.

(1) $$water\;absorption=\frac{m_{i}-m_{0}}{m_{0}}\;\cdot \;100\%,$$

where *m_i_*—mass of the sample after the immersion in water [g].

## 4. Conclusions

In this work, we proposed the method for development of algae biomass waste management by preparation of novel bio-based thermoplastic polyurethane elastomers composites. Three sets of composites were prepared and investigated in order to determine their chemical structure and selected thermal, mechanical, and physicochemical properties. By the usage of FTIR technique, the main functional groups present in the fillers and bio-TPU were described. Optic microscopy revealed the distribution of algae biomass waste and the tendency for filler agglomeration related to the filler shape. *Chlorella vulgaris*’ particles were fairly regular, spherical in shape but with the tendency to agglomerate formation. In the case of the *Enteromorpha–Zostera marina* blend, filler contains irregular, elongated fragments. Based on TGA analysis, it was determined that algae biomass wastes did not affect the thermal stability of the bio-TPU–ABW composites, which is advantageous. Bio-TPU matrices and ABW-based composites degrade in two main steps, mainly related to the segment structure of the bio-TPU matrix. DMA analysis revealed that the addition of algae biomass wastes had a negligible effect on the glass transition temperature of soft segments of bio-TPU–ABW composites, but influenced the loss factor. Tensile properties depend on the ABW type. Slight improvement in tensile strength and decrease in elongation at break was observed for S1 and S2 composite series. With increase in algae-based fillers, an increase in hardness was noticed. Water absorption was related to the type of algae-based filler. Composites filled with *Enteromorpha–Zostera marina* blend were characterized by almost twice as high water absorption rate (ca. 8% for the composites contained 15% of filler). Obtained results indicated the possibility to produce the new class of bio-TPU–ABW composites characterized by good thermal stability and mechanical properties with potential for their future application.

## Figures and Tables

**Table 1:** Parameters determined from the TG and DTG curves for algae-based fillers, bio-TPU matrices and bio-TPU–ABW composites.

Series	Code of Sample	T_5%_ (°C)	T_10%_ (°C)	T_50%_ (°C)	T_DTG1_ (°C)	T_DTG2_ (°C)	Residue at 600 °C (%)
S1	Filler_S1	143.1	248.1	568.1	263.7	319.8	49.2
	TPU	296.8	309.9	410.2	328.7	424.8	3.16
	S1_1	293.6	306.0	413.9	318.6	458.5	3.60
	S1_5	295.4	307.1	416.1	318.7	455.6	6.42
	S1_15	290.3	306.9	416.4	362.6	453.1	8.92
S2	Filler_S2	220.8	263.3	360.8	322.3	-	29.5
	TPU	296.8	309.9	410.2	328.9	423.9	3.16
	S2_1	290.4	303.4	410.7	321.3	459.2	4.15
	S2_5	299.1	313.0	414.7	329.8	460.9	4.26
	S2_15	295.4	311.7	417.1	330.2	457.2	6.04
S3	Filler_S3	223.5	263.6	358.6	322.2	-	29.0
	TPU	294.6	307.3	409.2	326.1	422.9	3.16
	S3_1	295.6	307.9	410.0	322.5	459.7	3.74
	S3_5	299.4	312.7	413.2	362.8	458.7	4.15
	S3_15	295.9	312.9	419.3	363.8	456.8	6.00

**Table 2:** Dynamic mechanical properties of bio-TPU matrixes and bio-TPU–ABW composites.

Series	Code of Sample	T_gSS_ (°C)	E’ (MPa)	E’ at 25 °C (MPa)	E” (MPa)	tanδ (-)
S1	TPU	−25.29	2343	46	236	0.375
	S1_1	−25.55	2321	42	219	0.372
	S1_5	−24.84	2382	46	225	0.385
	S1_15	−23.97	2326	75	220	0.328
S2	TPU	−25.41	2351	46	236	0.375
	S2_1	−25.98	2488	42	231	0.380
	S2_5	−25.41	2441	54	237	0.366
	S2_15	−23.31	2418	67	237	0.344
S3	TPU	−25.29	2361	51	236	0.370
	S3_1	−24.76	2102	50	200	0.360
	S3_5	−24.74	2406	61	228	0.357
	S3_15	−24.81	2385	68	234	0.330

**Table 3:** Tensile strength (**TS_b_**), elongation at break (**ε_Break_**) and hardness determined for bio-TPU matrices and bio-TPU–ABW composites.

Series	Code of Sample	TS_b_ (Mpa)	ε_Break_ (%)	Hardness (°ShA)
S1	TPU	2.85 ± 0.04	174 ± 8	71.5 ± 0.5
	S1_1	3.38 ± 0.08	243 ± 13	71.6 ± 0.5
	S1_5	2.58 ± 0.02	139 ± 11	62.2 ± 1.0
	S1_10	2.97 ± 0.06	107 ± 11	75.8 ± 0.8
	S1_15	2.49 ± 0.04	27 ± 3	72.9 ± 0.6
S2	TPU	2.44 ± 0.04	115 ± 7	72.8 ± 0.6
	S2_1	3.54 ± 0.10	244 ± 12	73.8 ± 0.5
	S2_5	2.76 ± 0.04	94 ± 7	76.6 ± 0.6
	S2_10	2.87 ± 0.07	109 ± 20	78.4 ± 0.3
	S2_15	2.89 ± 0.04	84 ± 5	79.4 ± 0.5
S3	TPU	2.97 ± 0.02	111 ± 4	73.4 ± 0.9
	S3_1	2.43 ± 0.02	74 ± 4	73.5 ± 0.9
	S3_5	2.18 ± 0.02	42 ± 2	76.1 ± 0.4
	S3_10	2.14 ± 0.03	36 ± 1	77.9 ± 0.2
	S3_15	2.21 ± 0.05	33 ± 1	78.4 ± 0.7

**Table 4:** Sample description: codes, series and content of the bio-components in the composites.

Sample Code	Series of Composites	Type of Algae	Algae Content wt.%	Content of the Bio-Components in the Composite
TPU	Matrix	-	0	68.7
S1_1	Series 1	Blend of seagrass *Zostera marina* and *Enteromorpha*	1	69.9
S1_5			5	71.0
S1_10			10	72.3
S1_15			15	73.5
S2_1	Series 2	Algae waste based on *Chlorella vulgaris* after algal oil extraction	1	69.9
S2_5			5	71.0
S2_10			10	72.3
S2_15			15	73.5
S3_1	Series 3	*Chlorella vulgaris* before algal oil extraction (reference filler)	1	69.9
S3_5			5	71.0
S3_10			10	72.3
S3_15			15	73.5

## Data Availability

Not applicable.

## Sample Availability

Samples of the compounds are not available from the authors.
